# Quantifying convergence on health-related indicators of the 2030 agenda for sustainable development

**DOI:** 10.2471/BLT.19.245811

**Published:** 2021-01-21

**Authors:** Fabrício Silveira, Ana Luísa Martins, Paulo Gadelha, Rômulo Paes-Sousa

**Affiliations:** a Fiocruz Minas, Grupo de Pesquisa em Políticas de Saúde e Proteção Social, Rua Uberaba, 780 sl. 5 subsolo. Cep. 30180-080, Belo Horizonte, Minas Gerais, Brazil.; b Fiocruz, Estratégia Fiocruz para a Agenda 2030, Rio de Janeiro, Brazil.

## Abstract

The extended scope and complexity of the United Nations 2030 agenda entail important challenges for the operationalization of the health-related sustainable development goal (SDG) indicators. Divergences in concepts, agendas and implementation strategies among institutions have fostered the parallel development of alternative and concurrent indicators. We aim to determine the convergences and divergences between five key institutions: the Global Burden of Disease Study (GBD), the Pan American Health Organization, the Sustainable Development Solutions Network, the World Bank and the World Health Organization (WHO). Of the 104 health-related indicators listed by these five institutions, 60 are consistent with official Inter-agency and Expert Group SDG indicators. Our analysis considers the indicators included, and the themes these indicators cover, in each institution list and each institution online platform. We quantified convergence in indicators between the institutions themselves, but also between the institutions and the official Inter-agency and Expert Group. Our results indicate important divergences; only 22 of the 60 indicators are included in the lists of all five institutions. The level of adoption of the official metrics varies from 40.5% (15/(47−10)) for the GBD to 86.2% (25/(29−0)) for the World Bank. WHO, the official curator of the Inter-agency and Expert Group SDG indicators, is only convergent with the official metrics by 72.1% (31/(45−2)). Our analysis, and the resulting awareness of the differences, potentialities and limitations of indicators and platforms, provides important contributions to enable the achievement of the health-related SDGs and deliver the promise of the 2030 agenda.

## Résumé

La complexité et l'envergure de l'Agenda 2030 des Nations Unies représentent d'importants défis pour la concrétisation des indicateurs régissant l'objectif de développement durable (ODD) visant à assurer la santé et le bien-être de tous. Les divergences de concepts, de programmes et de stratégies de mise en œuvre au sein des institutions ont engendré l'apparition simultanée d'indicateurs alternatifs et concurrents. Nous cherchons à identifier les convergences et divergences entre cinq institutions clés: l'étude sur la charge mondiale de morbidité (GBD), l'Organisation panaméricaine de la Santé, le Réseau des solutions pour le développement durable, la Banque mondiale et l'Organisation mondiale de la Santé (OMS). Sur les 104 indicateurs de santé repris par ces cinq institutions, 60 sont compatibles avec ceux du Groupe d'experts interinstitutionnel sur les indicateurs des ODD. Notre analyse tient compte des indicateurs inclus, ainsi que des domaines dont ils traitent, dans chaque liste institutionnelle et sur chaque plateforme institutionnelle en ligne. Nous avons quantifié la convergence d'indicateurs entre les institutions elles-mêmes, mais aussi entre ces institutions et le Groupe d'experts interinstitutionnel officiel. Nos résultats révèlent d'importantes divergences; seulement 22 des 60 indicateurs figurent dans les listes des cinq institutions. Le niveau d'adoption des paramètres officiels varie de 40,5% (15/(47–10)) pour le GBD à 86,2% (25/(29–0)) pour la Banque mondiale. L'OMS, administratrice officielle du Groupe d'experts interinstitutionnel sur les indicateurs des ODD, ne s'aligne sur les paramètres officiels qu'à 72,1% (31/(45–2)). Notre analyse et sa mise en évidence des différences, des possibilités et des limites des indicateurs et plateformes, contribue grandement à progresser vers la réalisation des ODD en matière de santé, et vers la concrétisation des promesses de l'Agenda 2030.

## Resumen

El amplio alcance y la complejidad del programa de las Naciones Unidas para 2030 implica importantes desafíos para la puesta en práctica de los indicadores del objetivo de desarrollo sostenible (ODS) relacionado con la salud. Las divergencias en los conceptos, los programas y las estrategias de aplicación entre las instituciones han fomentado la elaboración paralela de indicadores alternativos y simultáneos. Nos proponemos determinar las convergencias y divergencias entre cinco instituciones clave: el Estudio sobre la Carga Mundial de la Enfermedad (GBD, por sus siglas en inglés), la Organización Panamericana de la Salud, la Red de Soluciones para el Desarrollo Sostenible, el Banco Mundial y la Organización Mundial de la Salud (OMS). De los 104 indicadores relacionados con la salud enumerados por estas cinco instituciones, 60 son coherentes con los indicadores oficiales del ODS del Grupo Interinstitucional y de Expertos. Nuestro análisis tiene en cuenta los indicadores incluidos, y los temas que cubren estos indicadores, en cada lista de instituciones y en la plataforma en línea de cada institución. Cuantificamos la convergencia en los indicadores entre las propias instituciones, pero también entre las instituciones y el Grupo Interinstitucional y de Expertos oficial. Nuestros resultados indican importantes divergencias; solo 22 de los 60 indicadores están incluidos en las listas de las cinco instituciones. En cuanto al nivel de adopción de los indicadores oficiales varía entre el 40,5% (15/(47-10)) en el caso del GBD y el 86,2% (25/(29-0)) en el caso del Banco Mundial. La OMS, la comisaria oficial de los indicadores del ODS del Grupo Interinstitucional de Expertos, solo converge con los parámetros oficiales en un 72,1% (31/(45-2)). Nuestro análisis, y la consiguiente conciencia de las diferencias, potencialidades y limitaciones de los indicadores y plataformas, aporta importantes contribuciones para permitir el logro de los ODS relacionados con la salud y cumplir con el compromiso del programa de 2030.****

## ملخص

ينطوي كل من النطاق والسياق الموسعين لخطة الأمم المتحدة لعام 2030 على تحديات هامة لتفعيل مؤشرات أهداف التنمية المستدامة (SDG) المتعلقة بالصحة. وقد أدت الاختلافات في المفاهيم، وجداول الأعمال، واستراتيجيات التنفيذ بين المؤسسات، إلى تعزيز التطوير الموازي للمؤشرات البديلة والمتزامنة. نحن نهدف إلى تحديد أوجه التقارب والاختلاف بين خمس مؤسسات رئيسية: دراسة العبء العالمي للأمراض (GBD)، ومنظمة الصحة للبلدان الأمريكية، وشبكة حلول التنمية المستدامة، والبنك الدولي، ومنظمة الصحة العالمية (WHO). من بين 140 من المؤشرات المتعلقة بالصحة المدرجة في هذه المؤسسات الخمس، هناك 60 مؤشرًا تتسق مع مؤشرات أهداف التنمية المستدامة الرسمية المشتركة بين الوكالات ومجموعة الخبراء. يضع تحليلنا في الاعتبار المؤشرات المضمنة، والموضوعات التي تغطيها هذه المؤشرات، في قائمة كل مؤسسة وكل منصة على الإنترنت لكل مؤسسة. قمنا بقياس التقارب في المؤشرات بين المؤسسات نفسها، ولكن أيضًا بين المؤسسات ومجموعة الخبراء الرسمية المشتركة بين الوكالات. تشير نتائجنا إلى اختلافات مهمة؛ تم تضمين 22 مؤشرًا فقط من بين 60 مؤشرًا في قوائم كل المؤسسات الخمس. يختلف مستوى تطبيق المقاييس الرسمية من 40.5% (15/(47-10)) للعبء العالمي للأمراض إلى 86.2% (25/(29–0)) للبنك الدولي. منظمة الصحة العالمية، التي تمثل المنسق الرسمي لمؤشرات أهداف التنمية المستدامة لمجموعة الخبراء المشتركة بين الوكالات، تتقارب فقط مع المقاييس الرسمية بنسبة 72.1% (31/(45-2)). إن تحليلنا، والوعي الناتج عن اختلافات وإمكانيات وقيود المؤشرات والمنصات، يوفر مساهمات مهمة لتمكين تحقيق أهداف التنمية المستدامة المتعلقة بالصحة والوفاء بوعد أجندة العمل لعام 2030.

## 摘要

联合国《2030 年可持续发展议程》的范围不断扩大且复杂度越来越高，这给健康相关可持续发展目标 (SDG) 指标的实现带来了重大挑战。各机构在概念、议程和执行战略方面的差异促使各机构同步制定了替代指标和并行指标。我们旨在确定五个关键机构之间的趋同与分化：全球疾病负担研究 (GBD)、泛美卫生组织、可持续发展解决方案网络、世界银行和世界卫生组织 (WHO)。在这五个机构列出的 104 个健康相关指标中，有 60 个与官方的机构间专家组 SDG 指标一致。我们分析时考虑了各机构列表和各机构在线平台中包含的指标以及这些指标涵盖的主题。我们不仅对上述机构之间的指标趋同度进行量化，还对这些机构与官方机构间专家组间的指标趋同度进行了量化。我们的结果显示存在重大分化；这五个机构的列表仅包括了 60 个指标中的 22 个。官方指标的采纳率从 GBD 的 40.5% (15/(47–10)) 至世界银行的 86.2% (25/(29–0)) 不等。世卫组织是机构间专家组 SDG 指标的官方监管组织，与官方指标的趋同率仅为 72.1% (31/(45–2))。我们的分析以及由此产生的对指标和平台的差异、潜力和局限性的认识，为实现健康相关 SDG 和履行《2030 年可持续发展议程》的承诺做出了重要贡献。

## Резюме

Расширенный охват и сложность повестки дня Организации Объединенных Наций на период до 2030 года влечет за собой серьезные проблемы с практическим внедрением показателей состояния здоровья для целей в области устойчивого развития (ЦУР). Расхождения в концепциях, повестках дня и стратегиях реализации между учреждениями способствовали параллельной разработке альтернативных и параллельных показателей. Авторы стремятся определить совпадения и расхождения между пятью ключевыми учреждениями: исследованием глобального бремени заболеваний (ГБЗ), Панамериканской организацией здравоохранения, сетью решений для обеспечения устойчивого развития, Всемирным банком и Всемирной организацией здравоохранения (ВОЗ). Из 104 показателей состояния здоровья, указанных этими пятью учреждениями, 60 соответствуют официальным показателям межучрежденческой и экспертной группы по показателям достижения целей в области устойчивого развития. В нашем анализе рассматриваются включенные показатели и темы, которые охватывают эти показатели, в списке и на онлайн-платформе каждого учреждения. Авторы выполнили количественную оценку сходства показателей как между самими учреждениями, так и между учреждениями и официальной межучрежденческой и экспертной группой. Наши результаты указывают на важные расхождения. Только 22 из 60 показателей включены в списки всех пяти учреждений. Уровень принятия официальных показателей варьируется от 40,5% (15/(47–10)) для ГБЗ до 86,2% (25/(29–0)) для Всемирного банка. Значения ВОЗ, официального куратора показателей межучрежденческой и экспертной группы по показателям достижения целей в области устойчивого развития, совпадают с официальными показателями лишь на 72,1% (31/(45–2)). Наш анализ и вытекающая из этого осведомленность о различиях, возможностях и ограничениях показателей и платформ вносят важный вклад в достижение ЦУР, связанных со здоровьем, и выполнение обещаний повестки дня на период до 2030 года.

## Introduction

The United Nations (UN) 2030 agenda for sustainable development is organized into 17 complex and interdependent sustainable development goals (SDGs), encompassing 169 targets related to aspects of socioeconomic and environmental determination. The official SDGs framework is described by 231 globally harmonized monitoring indicators, designed by the Inter-agency and Expert Group to enable multilevel global comparisons across time and avoid misinterpretation of the targets.^1^ The Inter-agency and Expert Group also has the official mandate for establishing and periodically reviewing the official indicator framework for the SDGs, in addition to monitoring the global coverage of these indicators.^2^

However, regardless of the establishment of globally harmonized monitoring indicators, divergences in concepts, agendas and implementation strategies among the institutions promoting the SDGs exist. Further, the methodological challenges brought by newly developed indicators and a lack of official and/or comparable data for many countries have fostered the parallel development of alternative and concurrent indicators.^3^ Although this parallel development has led to an increase in the number of countries and subnational units working towards the 2030 agenda,^3^ such a nonuniform approach can reduce the capacity of the agenda to effect changes at the global scale; indeed, this competitive rather than collaborative environment among the stakeholders contradicts the very principles of the 2030 agenda.^4^

**Fig. 1:**
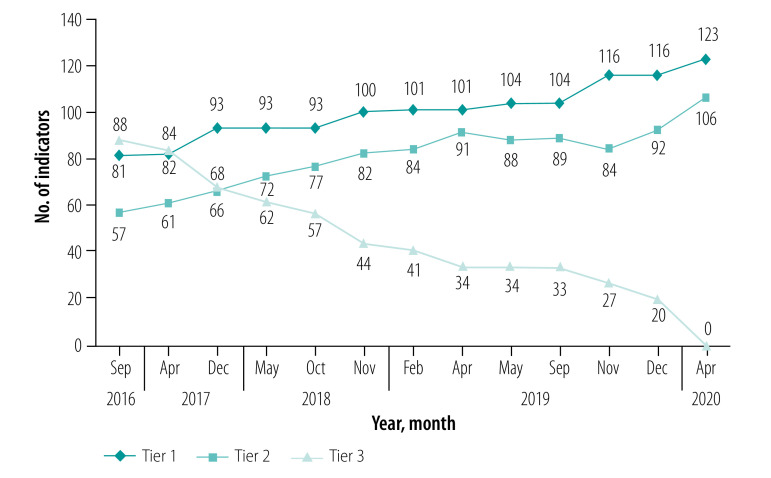
Progress in SDG indicator operationalization in terms of tier classification, 2016–2020

The level of progress in official SDG indicator operationalization is represented by its tier classification. Currently, 53.7% (123/229) of the indicators are classed as Tier 1, that is, “conceptually clear, has an internationally established methodology and standards are available, and data are regularly produced by countries for at least 50 per cent of countries and of the population in every region where the indicator is relevant”.^5^ Since the last revision in April 2020, there are no indicators currently classed as Tier 3 (Fig. 1), for which “no internationally established methodology or standards are yet available for the indicator, but methodology/standards are being (or will be) developed or tested”.^5^ However, the fact that 45.9% (106/231) of the indicators are currently classified as Tier 2 – “conceptually clear, has an internationally established methodology and standards, but data are not regularly produced by countries”^5^ – highlights how the availability of comparable data is the biggest challenge for statistical agencies worldwide. This challenge reinforces the UN global call for governments to improve statistical capacity and data collection, strengthen cooperation and international partnerships, and agree on collective action to deal with the complexity and interdependence of planetary demands.^3^^,^^7^

SDG: sustainable development goal.

Note: Tier 1 represents indicators that are conceptually clear, where internationally established methodology and standards are available, and data are regularly produced by countries for at least 50% of countries and of the population in every region where the indicator is relevant. Tier 2 represents indicators that are conceptually clear, have an internationally established methodology and standards, but data are not regularly produced by countries. Tier 3 represents indicators where no internationally established methodology or standards are yet available for the indicator, but methodology/standards are being (or will be) developed or tested. Graph does not include indicators that were classified as multitier.

Data source: Kronemberger^6^ (2016–2017 data) and Inter-agency and Expert Group SDG tier classification reports (2018–2020).^5^

In this paper, we aim to determine the convergences and divergences in the operationalization of the health-related SDG indicators according to five key global institutions – the Global Burden of Disease Study (GBD), the Pan American Health Organization (PAHO), the Sustainable Development Solutions Network, the World Bank and the World Health Organization (WHO) – selected for inclusion in our analysis for their international prestige and pre-eminent role in 2030 agenda initiatives. By understanding where the aims of these institutions diverge and converge, we provide important insights for the strategic organization of the international community in the achievement of the 2030 health targets.

## Comparison methods

The five institutions that we consider here, listed in Box 1, have selected and adapted a set of health-related indicators based on their area of expertise, statistical capacity, traditions and views on health determinants. Of the 104 health-related indicators listed by these five institutions, 60 are consistent with official Inter-agency and Expert Group SDG indicators. In addition to the 13 targets and 27 indicators included in SDG 3 (ensuring a healthy life and promoting well-being at all ages), the five institutions also consider a further 33 health-related indicators included in SDGs 1 (poverty), 2 (hunger), 4 (education), 5 (gender equality), 6 (sanitation), 7 (energy), 10 (inequalities), 11 (sustainability), 16 (strong institutions) and 17 (partnerships).

Box 1 **Institutions included in analysis of quantifying convergence in health-related SDG indicators, 2020**

**Global Burden of Disease Study**

International consortium of more than 3600 researchers from 145 countries, led by the Institute for Health Metrics and Evaluation of the University of Washington, United States of America. The institution collects data and develops estimates for disabilities, diseases, injuries and risk factors.^8^

**Pan American Health Organization**

Created in 1902, it is the oldest health organization in the world. It currently has 52 member countries and two simultaneous institutional functions, being both the specialized health agency of the Inter-American System and the Regional Office for the Americas of WHO.^9^

**Sustainable Development Solutions Network**

This independent global network of 800 member institutions, including technical institutions, research centres and universities, aims to mobilize available technical knowledge to resolve sustainable development issues. It received a UN mandate in 2012 to support the 2030 agenda.^10^

**World Bank**

This independent specialized agency of the UN acts as a major financier of developing countries, with 12 000 projects to support world development.^11^

**WHO**

The UN’s specialized health agency comprising 194 member countries.^12^

SDG: sustainable development goal; UN: United Nations; WHO: World Health Organization.

Our analysis considers the indicators included, and the themes these indicators cover, in each institution list and each institution online platform. We compared indicators between the institutions themselves, but also between the institutions and the official Inter-agency and Expert Group. We also compared the methodological choices made by the institutions in operationalizing the indicators, that is, the convergence in indicators with the official indicators as designed by the Inter-agency and Expert Group. For example, although the Sustainable Development Solutions Network included the indicator for malnutrition among children younger than 5 years in their list, their platform shows obesity among adults as the indicator; this means that this particular indicator is not considered to be convergent with the official metrics.

The SDG information platforms provided by the various institutions are the main tools for converting the 2030 agenda to policies. These platforms aim to fill analytical gaps by focusing on different perspectives of the tasks of promoting, monitoring and evaluating the challenges of the 2030 agenda, that is, operationalizing their institution’s health-related SDG indicators. We therefore also assessed the general characteristics of the platforms provided by the five institutions. In comparing these platforms, we considered: (i) temporal and geographical coverage of the database; (ii) indicators (i.e. whether official SDG indicators or alternatives resulting from an institution-specific interpretation); (iii) methods of presentation and dissemination (e.g. visual aids, reports); and (iv) methods of global assessment (e.g. international comparisons, temporal analysis, performance indices, trends and projections).

## Convergences and divergences

### Themes

We illustrate the thematic convergence between the institutions in Table 1, adopting the thematic classification for the health-related indicators devised by WHO for themes.^13^ We devised an additional category, that is other themes to reflect new topics introduced by the other institutions. As anticipated, due to the number of inherited indicators from the millennium development goals (MDGs), the themes reproductive and maternal health, newborn and child health, infectious diseases, and noncommunicable diseases are relatively consensual. Themes on injuries and violence and environmental risks include a larger number of indicators, explaining the slightly lower levels of agreement between the institutions than for themes inherited from the MDGs. The least consensual themes (as measured by the number of indicators in the theme adopted by the institutions) are: universal health coverage and health systems, which contains indicators that have been less studied in recent years due to the presence of a vertical disease approach to health interventions;^14^ and other themes, which includes five new topics. 

**Table 1:** Convergence on official health-related SDG indicators operationalized by five key global institutions, 2020

Indicator included by theme	No. Inter-agency and Expert Group SDG indicators (indicator no.)	No. of indicators				
		GBD	PAHO	Sustainable Development Solutions Network	World Bank	WHO
**Reproductive and maternal health**	6 (3.1.1, 3.1.2, 3.7.1, 3.7.2, 5.6.1, 5.6.2)	6	3	3	4	6
**Newborn and child health**	5 (2.2.1, 2.2.2, 3.2.1, 3.2.2, 3.b.1)	5	5	5	5	5
**Infectious diseases**	5 (3.3.1–3.3.5)	5	5	5	4	5
**Noncommunicable diseases**	5 (3.4.1, 3.4.2, 3.5.1, 3.5.2, 3.a.1)	5	5	4	4	5
**Injuries and violence**	12 (1.5.1/11.5.1/13.1.1, 3.6.1, 5.2.1, 5.2.2, 5.3.1, 5.3.2, 8.8.1, 16.1.1–16.1.4, 16.2.3)	10	6	5	1	8
**Environmental risks**	10 (3.9.1–3.9.3, 4.a.1, 6.1.1, 6.2.1, 6.3.1, 6.a.1, 7.1.2, 11.6.2)	8	4	7	5	8
**UHC and health systems **	8 (1.a.2, 3.8.1, 3.8.2, 3.b.2, 3.b.3, 3.c.1, 3.d.1, 17.19.2)	7	6	4	4	8
**Other**						
Social protection	1 (1.3.1)	0	0	1	0	0
Poverty	2 (1.1.1, 2.1.1)	0	0	1	1	0
Infrastructure	1 (7.1.1)	0	0	1	0	0
Migration	2 (10.7.1, 10.7.2)	0	2	0	0	0
Statistics and data production	3 (16.9.1, 17.18.1, 17.18.2)	1	2	1	1	0
**Total no. health-related indicators on lists**	60	47	38	37	29	45

GBD: Global Burden of Disease Study; PAHO: Pan American Health Organization; SDG: sustainable development goal; UHC: universal health coverage; WHO: World Health Organization.

### Indicators and their metrics

**Fig. 2:**
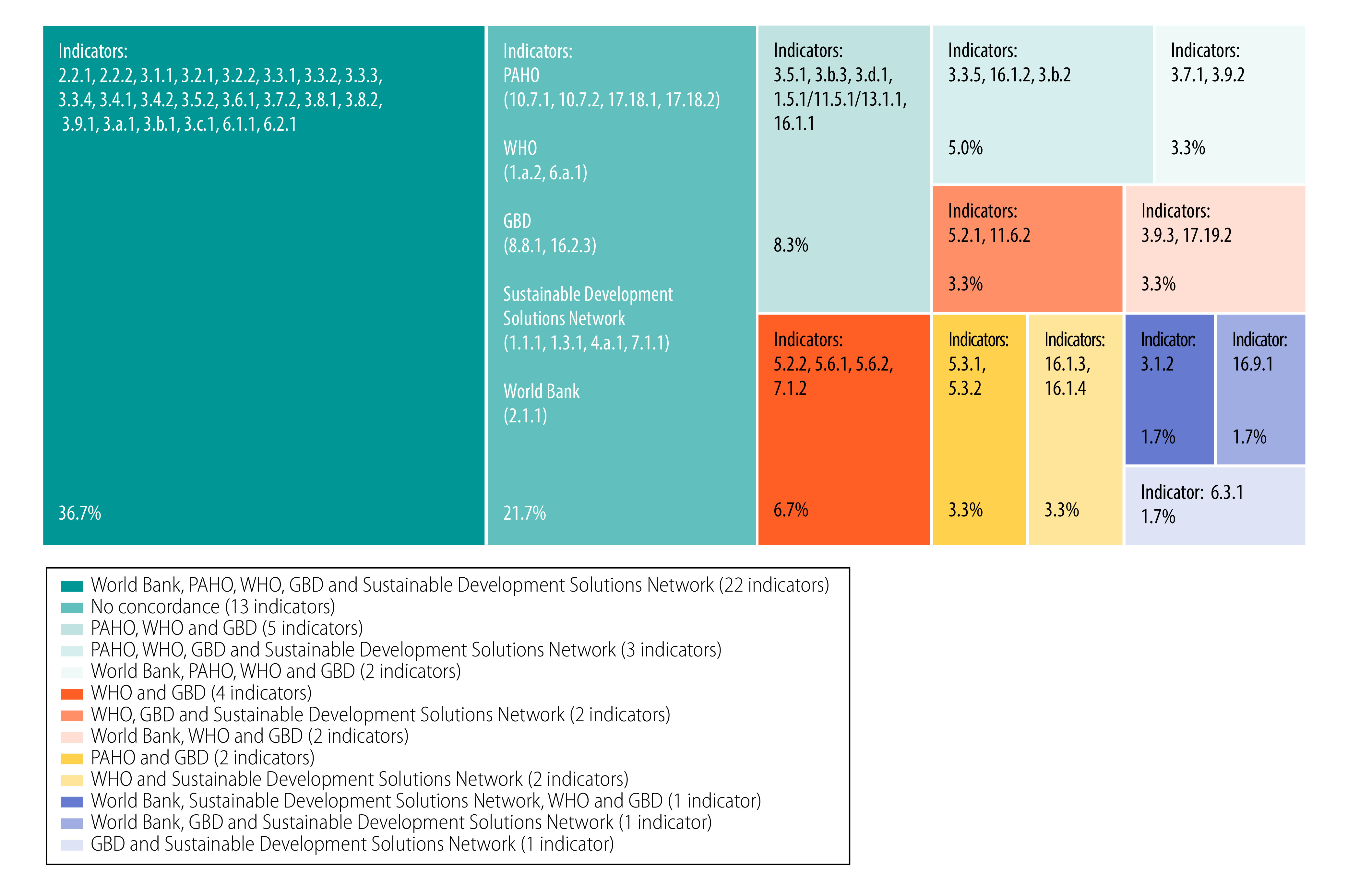
Convergence in health-related SDG indicators between five key institutions, 2020

GBD: Global Burden of Disease Study; PAHO: Pan American Health Organization; SDG: sustainable development goal; WHO: World Health Organization.

We depict the convergences between the five institutions in terms of indicators included in Fig. 2. Of the 60 health-related SDGs considered, 47 (78.3%) of these are included in the lists of at least two institutions. Only 22 of the 60 indicators (36.7%) were included in the lists of all five institutions. The second-largest group in Fig. 2 (indicated by the polygon area) comprises 13 indicators (21.7%) that are only included by a single institution. The third-largest group comprises five indicators agreed upon only by the GBD, PAHO and WHO. The fourth-largest group represents four indicators agreed upon only by the GBD and WHO, which are the two institutions with the greatest level of synergy in their choices. The other nine polygons represent one to three indicators covered by different pairs of institutions.

Table 2 shows a summary of the number of indicators considered between each individual institution and the Inter-agency and Expert Group; a more detailed table is available from the data repository.^22^ For each institution, we define the level of adoption of the official metrics as the number of convergent indicators as a percentage of the number of health-related indicators less the number of non-operationalized indicators. The level of adoption of the official metrics varies from 40.5% (15/(47−10)) for the GBD to 86.2% (25/(29−0)) for the World Bank. WHO, the official curator of the Inter-agency and Expert Group SDG indicators, is only convergent with the official metrics by an amount of 72.1% (31/(45−2)).

**Table 2:** Comparison of institute platforms for presentation and assessment of health-related SDG indicators, 2020

Characteristic	GBD	PAHO	Sustainable Development Solutions Network	World Bank	WHO	UN Department of Economic and Social Affairs
Title of platform	Health-related SDGs^15^	Health information platform for the Americas^16^	Sustainable Development Report^17^^,^^18^	World Bank Open Data^19^	World Health Data Platform^20^	Global SDG Indicators Database^21^
Coverage						
No. countries	188	34	156	217	> 190	260
Period	1990–2030	1989–2018^a^	2000–2018	1990–2019^a^	1989–2019^a^	2000–2019^a^
No. SDG indicators in list	47	38	37	29	45	–
Non-operationalized	10	16	14	0	2	–
No. SDG indicators on platform	37	27	30	43	46	56^b^
Convergent	15	14	18	25	31	–
Expanded	44	38	35	61	68	123
Level of adoption of official metrics (%)^c^	40.5	63.6	78.3	86.2	72.1	–
Methods of presentation	Cartograms, raw data, line/radar graphs, scatter plots	Cartograms, raw data, bar/line graphs, scatter plots	Cartograms, bar graphs	Raw data, line graphs, reports	Cartograms, raw data, bar/line graphs, scatter plots^d^	Raw data
Methods of assessment	Historical indicator evolution, performance index, projections	Historical indicator evolution, ranking of units, trends	Performance index, trends	Historical indicator evolution	Ranking of units	–

GBD: Global Burden of Disease Study; PAHO: Pan American Health Organization; SDG: sustainable development goal; UN: United Nations; WHO: World Health Organization.

^a^ Cross-section, period coverage, presentation and graphical features vary with the indicators.

^b^ 123 indicators included, but 67 correspond to either alternative indicators for different dimensions of the target (e.g. 4.a.1) or versions of the same indicator with totals and percentages (e.g. 2.2.2).

^c^ No. convergent indicators as a percentage of the number of health-related indicators less the number of non-operationalized indicators.

^d^ Only available in raw data format for downloading.

### Platforms 

We summarize the main characteristics of the platforms of the five institutions, plus the official UN dissemination platform, in Table 2. For concision, we only reference the websites of the main platform of each institution (some may host more than one, providing individual websites for data visualization, material for promoting and/or evaluating the SDGs, or topic-specific solutions).

The platforms demonstrate different methodological perspectives and therefore occupy distinct, albeit complementary, roles in achieving the 2030 agenda. The platforms of the Sustainable Development Solutions Network and WHO highlight the latest data and provide international comparisons. The platforms of GBD, PAHO and the World Bank focus on the evolution of indicators, either for individual countries or in international comparisons. In terms of critical assessments of progress in achieving the 2030 agenda targets, the Sustainable Development Solutions Network platform provides measures of achievement of targets and goals, while the GBD platform provides projections for the next decade. 

Table 2 also describes the inconsistencies between the indicators included in the lists of institutions and those made available on their platforms. The number of operationalized indicators is less than the number of listed indicators for three of the institutions (GBD, PAHO and the Sustainable Development Solutions Network), indicating differences between aims and what is possible to put into practice. In contrast, the World Bank and WHO appear to have a higher number of indicators on their platforms than in their lists, as a result of deriving several platform indicators from a single list indicator; we also provide this expanded number of platform indicators in Table 2. 

The GBD platform^15^ provides interactive time-series visualizations using data constructed by their own consortium.^8^ Although this platform stands out for presenting year-to-year projections until 2030 (disaggregated by age group and sex in most cases), the methods of data collection, use of sampling techniques, interpolation and eventual estimation of missing data have attracted criticism regarding their validity and consistency.^23^

The PAHO platform^16^ is an interactive dashboard with good graphical visualization of the indicators and a diversity of resources (e.g. international rankings, trends, country profiles and maps). The dashboard also enables the disaggregation of the data into various domains (e.g. demographic, economic, health care, risk factors, coverage and status) and subdomains (causes and mortality).

The Sustainable Development Solutions Network offers three integrated platforms to monitor and evaluate the SDG indicators. The first brings together annual reports produced from 2016 onwards with the aim of complementing the official SDG indicators and voluntary reports from member countries.^17^ The second is an interactive dashboard that provides international ranks and country profiles that highlight the national performance and trend. Both initiatives are based on the institution’s SDG index, calculated using official and unofficial data for variables distinct from those in the official indicators. The most recently launched (July 2020) platform^18^ focuses on real-time SDG indicators.

The World Bank provides several platforms for monitoring and assessing SDG indicators, including an atlas and dashboard. Powered with data from the World Bank’s officially recognized World Development Indicators, it provides a vast list of indicators for all SDGs.^19^ The historical series for any given indicator may span many decades, reflecting the institution’s long tradition of international data compilation.

WHO’s platform,^20^ part of the Global Health Observatory, provides global action plans, annual health monitoring reports, infographics and estimates of the costs of achieving health goals. Country-level data, in addition to maps and graphs, are also available for each indicator.

## Discussion

The process of formulating the global indicators to monitor progress towards the SDGs was influenced more by politics than technicalities.^24^ As a result, differences between the aspirational principles stated in the agenda and the actual monitoring indicators can be expected. Indeed, we observed four epistemological reductions (three qualitative and one quantitative) in the transition between the Rio Declaration (launched at the Rio+20 Conference)^25^ and the SDG indicators operationalized by the institutions. The first two reductions occurred during the process of converting the declaration to goals and targets as a result of the inevitable translation in terms of both language and anchorage (i.e. philosophical and political principles) in preparing tangible policies.^26^^,^^27^ The third qualitative reduction occurred as a result of the constraints of conceptional and algebraic representations of complex phenomena during the conversion of targets into official lists of indicators. The final (quantitative) reduction occurred when applying the indicators to available circumstances, that is, adapting the information in the benchmark Inter-agency and Expert Group SDG indicators into indicators consistent with the statistical traditions of the institutions and data availability. Our investigation focused on this final reduction, that is, the divergences and convergences between five key global health institutions in operationalized indicators.

Both the choice of health-related indicators, and whether operationalized indicators are convergent with official indicators, diverged significantly between the five institutions. This variety of aims increases the number of countries and regions working towards the 2030 agenda and also enhances discussion of the determinants of health. Indeed, this multidimensional nature can effectively contribute to a better conceptualization of public and global health. Including the views of governments and states on health-related themes and challenges, expressed in the Voluntary National Reviews,^28^ is also important.

As data become more ubiquitous, aiming at a higher level of consensus should be seen as a central condition to leveraging the application of SDGs globally for two reasons: (i) divergences generate discrepancies in assessments and (ii) consensus simplifies navigation of the complex 2030 agenda. Standardization in reporting increases comparability, sending a more direct message to policy-makers and society. Likewise, the prioritization of consensual indicators, upon which clear targets can be set, increases usability. Ultimately, establishing a clear roadmap to achievement of the SDGs could help to break resistance where necessary.

There has been much progress to date; important initiatives have been launched to mobilize more (and improved) data to monitor the SDGs coordinated by the UN Department of Economic and Social Affairs, the Sustainable Development Solutions Network and other global institutions and governments. On the health theme, we note that WHO has recently introduced new and more metric-convergent indicators: 10 new indicators were added to the institution’s platform during July 2019–October 2020. Over the same period, PAHO and the World Bank added three new health-related indicators while the UN Department of Economic and Social Affairs added five indicators.

A strength of our analysis is that we revealed some consolidated themes and indicators, but also large divergences between the institutions in terms of either the chosen set of indicators, operationalization metrics and the use of data in monitoring and evaluating progress. A limitation of our study is that it was not exhaustive; different statistical methods and/or sources of data (especially with the increasing use of nonofficial data as a result of their greater availability) for the SDG indicators can also contribute to divergences in the analysis between the institutions. Ultimately, more research is necessary to understand the impact of choosing one metric or platform over another in the monitoring of progress towards the SDGs.

As we enter the final decade of the 2030 sustainable development agenda, it is time to take decisive steps towards transforming data into action. This transformation requires that governments commit to greater adoption of the SDG framework, that is, incorporate SDGs into development programmes and increase efforts to implement policies targeting inequalities. Our analysis, and the resulting awareness of the differences, potentialities and limitations of indicators and platforms, provides important contributions to enable the relevant challenges to be met. The achievement of the health-related SDGs – for which the supply of timely and disaggregated information vital to the design and evaluation of adequate measures is essential – requires a data revolution at all levels. A coherent and unified view of global health challenges is necessary to achieve the SDGs and deliver the promise of the 2030 agenda.
